# Detection of peptide-specific CTL-precursors in peripheral blood lymphocytes of cancer patients

**DOI:** 10.1038/sj.bjc.6600548

**Published:** 2002-09-23

**Authors:** Y Maeda, N Hida, F Niiya, K Katagiri, M Harada, H Yamana, T Kamura, M Takahashi, Y Sato, S Todo, K Itoh

**Affiliations:** Department of Immunology, Kurume University, 67 Asahi-machi, Kurume, 830-0011, Japan; First Department of Surgery, Hokkaido University, N15-W7 Kita-ku, Sapporo 060-8638, Japan; Department of Surgery, Kurume University School of Medicine, 67 Asahi-machi, Kurume, 830-0011, Japan; Department of Obstetrics and Gynecology, Kurume University School of Medicine, 67 Asahi-machi, Kurume, 830-0011, Japan

**Keywords:** immunotherapy, cancer, peptide vaccine, CTL-precursors, pre-vaccination

## Abstract

Development of therapeutic vaccines is one of the major areas of tumour immunotherapy today. However, clinical trials of peptide-based cancer vaccines have rarely resulted in tumour regression. This failure might be due to an insufficient induction of cytotoxic T lymphocytes in the current regimes, in which cytotoxic T lymphocytes-precursors in pre-vaccination peripheral blood mononuclear cells are not measured. Initiation of immune-boosting through vaccination could be better than that of immune-priming with regard to induction of prompt and strong immunity. If this is also the case for therapeutic vaccines, pre-vaccination measurement of peptide-specific cytotoxic T lymphocytes-precursors will be important. In the present study, we investigated whether cytotoxic T lymphocytes-precursors reacting to 28 kinds of peptides of vaccine candidates (13 and 15 peptides for HLA-A24^+^ and HLA-A2^+^ patients, respectively) were detectable in pre-vaccination peripheral blood mononuclear cells of 80 cancer patients. Peptide-specific cytotoxic T lymphocytes-precursors were found to be detectable in peripheral blood mononuclear cells of the majority of cancer patients (57 out of 80 cases, 71%). The mean numbers of positive peptides were 2.0 peptides per positive case. Peripheral blood mononuclear cells incubated with positive peptides, not with negative peptides, showed significant levels of HLA-class-I-restricted cytotoxicity to cancer cells. The profiles of positive peptides entirely varied among patients, and were not influenced by the cancer origin. These results may provide a scientific basis for the development of a new approach to cancer immunotherapy, e.g.) cytotoxic T lymphocytes-precursor-oriented peptide vaccine.

*British Journal of Cancer* (2002) **87**, 796–804. doi:10.1038/sj.bjc.6600548 www.bjcancer.com

© 2002 Cancer Research UK

## 

Recent advances in molecular biology and tumour immunology have allowed for identification of a large number of genes and antigenic peptides recognised by cytotoxic T lymphocytes (CTLs), thereby introducing the possibility of a peptide-based cancer vaccine (Bruggen *et al*, 1991; Kawakami *et al*, 1994; Fisk *et al*, 1995; Correale *et al*, 1998). Preventive vaccine protocols for pathogenic microbes generally consist of three steps; priming, boosting and challenging (Amara *et al*, 2001). It usually takes several months for priming, which is consistent with the present results of our clinical trial using SART3-peptide vaccine (Miyagi *et al*, 2001). Although increased CTL activity has been obtained in the majority of cancer patients vaccinated with the peptides, clinical responses have rarely been obtained in these patients in either previous studies of melanoma patients (Rosenberg *et al*, 1998; Marchand *et al*, 1999; Wang *et al*, 1999; Gajewski *et al*, 2001), or our study of epithelial cancer patients (Miyagi *et al*, 2001).

One explanation for the failure to obtain clinical responses would be the time-lag needed for priming of anti-tumour response, given that the expected survival of most advanced cancer patients under these regimens is 6–9 months. Therefore, developing a new protocol for obtaining tumour regression in these cancer patients is necessary. One protocol might be a pre-vaccination measurement of peptide-specific CTL-precursors in the circulation of cancer patients, followed by administration of CTL-precursor-oriented peptide vaccine. This protocol may more rapidly increase CTL activity in post-vaccination peripheral blood mononuclear cells (PBMCs). Pre-vaccination measurement of CTL-precursors could also be an important factor in the generation of strong immune response, since Langerhans cells presenting peptides need to meet peptide-specific CTL-precursors within 2 days at the regional lymph nodes to activate CTLs (Janeway *et al*, 1999). Further, recent studies on memory CD8^+^T cells suggest that the main effect of vaccine boosters is to increase the number of antigen-specific memory T cells to one that confers better protection (Kaech and Ahmed, 2001; Finn and Lotze, 2001; Stipdonk *et al*, 2001).

In the present study, we investigated whether peptide-specific CTL-precursors are detectable in pre-vaccination PBMCs from cancer patients, and report herein that CTL-precursors reacting to cancer-related peptides are detectable in prevaccination PBMCs of the majority of cancer patients.

## MATERIALS AND METHODS

### Patients, cell lines, and peptides

PBMCs were isolated from 20 ml of heparinized blood of HLA–A24^+^ (*n*=55) and HLA–A2^+^ (*n*=25) cancer patients (40 gastric, 15 colon, 11 lung, seven gynaecological, three prostate cancers, and four melanomas) by means of Ficoll–Conray density gradient centrifugation as reported previously (Gomi *et al*, 1999). Among them, 56 patients had no distant metastases and 24 patients had distant metastases. No patient had undergone any immunotherapy or chemotherapy at least for 1 month before sampling. Complete informed consents were obtained from all patients. PBMCs were also obtained from 31 healthy volunteers (20 HLA–A24^+^ and 11 HLA–A2^+^). HLA-class-I typing was performed on blood lymphocytes using the classical serological method, as reported previously (Gomi *et al*, 1999). The tumour cell lines used in this study are as follows: KWS (HLA-A0206/0206), SSTW9 (A2402/2601) gastric adenocarcinoma, Panc-1 (A0201/0206) pancreatic adenocarcinoma, and SW620 (A0201/2402) colon adenocarcinoma. PHA-activated normal T cells were also used as target. Peptides used in this study are listed in Table 1

**Table 1:**
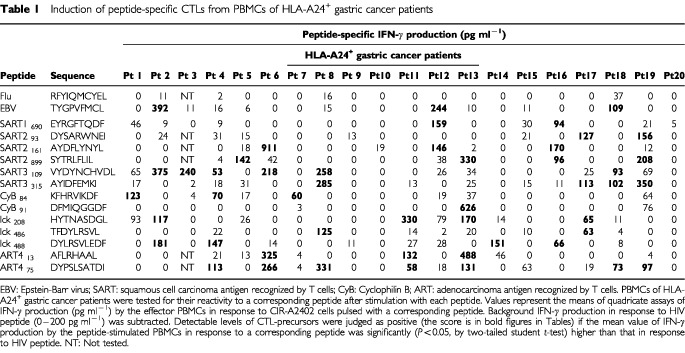
Induction of peptide-specific CTLs from PBMCs of HLA-A24^+^ gastric cancer patients

for HLA-A24-restricted peptides, and in Table 2

**Table 2:**
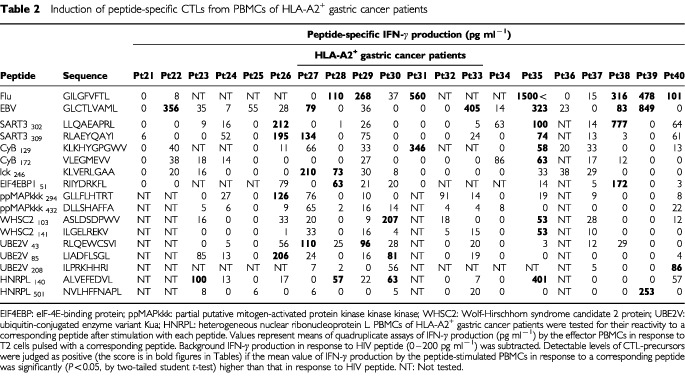
Induction of peptide-specific CTLs from PBMCs of HLA-A2^+^ gastric cancer patients

for HLA-A2-restricted peptides. All peptides except for Epstein–Barr virus (EBV)- and Influenza virus (Flu)-derived peptides are encoded by tumour-rejection antigens, and have the ability to induce HLA-A24 or -A2-restricted CTLs specific to tumour cells in PBMCs of cancer patients, as reported previously (Shichijo *et al*, 1998; Gomi *et al*, 1999; Yang *et al*, 1999; Ito *et al*, 2000, 2001; Kawano *et al*, 2000; Nakao *et al*, 2000; Nishizaka *et al*, 2000; Harashima *et al*, 2001; Tamura *et al*, 2001). These peptides were purchased from Sawady Laboratory (Tokyo, Japan), and their purity was >95%.

### Detection of CTL-precursors

A simple method was used to detect peptide-specific CTLs in PBMCs (Hida *et al*, 2002). PBMCs (1×10^5^ cells per well) were incubated with 10 μM of a peptide in wells of the u-bottom-type 96-well microculture plates (Nunc, Roskilde, Denmark) in 200 μl of culture medium. The culture medium consisted of 45% RPMI-1640 medium, 45% AIM-V® medium (GIBCO–BRL), 10% FCS, 100 U ml^−1^ of interleukin-2 (IL-2), and 0.1 μM MEM nonessential amino acid solution (GIBCO–BRL). Half of the medium was removed and replaced with the new medium containing a corresponding peptide (20 μM) every 3 days. After incubation for 12 days, these cells were harvested and then tested for their ability to produce IFN-γ in response to CIR-A2402 cells pre-loaded with either a corresponding peptide or HIV peptide (RYLRQQLLGI) as a negative control in HLA-A24^+^ PBMCs, or those in response to T2 cells pre-loaded with a corresponding or HIV peptide (SLYNTYATL) in HLA-A2^+^ PBMCs by an enzyme-linked immuno-sorbent assay (ELISA) (limit of sensitivity: 10 pg ml^−1^). All experiments were performed in quadricate assays. Detectable levels of CTL-precursors were judged as positive (the score is in bold in Tables) if the mean value of IFN-γ production by the peptide-stimulated PBMCs in response to a corresponding peptide was significantly (*P*<0.05) higher than that in response to control HIV peptide. A two-tailed Student *t*-test and Fisher's exact probability test were employed for the statistical analyses.

### Cyototoxicity assay of the peptide-induced CTLs

The peptide-stimulated PBMCs were further incubated in the presence of feeder cells for 3 weeks in order to obtain a relatively large number of cells, and were tested for their cytotoxicity against various target cells by a standard 6 h ^51^Cr-release assay. For an inhibition assay, the ^51^Cr-release assay was performed in the presence of 20 μg ml^−1^ of anti-CD8, -CD4, -HLA-class I (W6/32), or -HLA-class II (DR) mAb. A standard CTL precursor frequency analysis was performed in certain cases, and the detailed description of this method is reported elsewhere (Miyagi *et al*, 2001). Briefly, cells were incubated at 12.5, 25, 50, 100, 200 and 400 cells per well of 96-well microculture plate in the presence of feeder cells. Cells from each well were harvested and tested at 9 to 15 days of culture in duplicate assay for their ability to produce IFN-γ by recognition of peptide-pulsed target cells. The well was considered positive if it contained effector cells producing much higher levels (>100 pg ml^−1^) and also statistically significant levels (*P*<0.05 by Student *t*-test) of IFN-γ in response to CIR-A2402 cells (or T2 cells for HLA-A2 cases) pre-loaded with a corresponding peptide as compared with those in response to CIR-A2402 cells (or T2 cells) pre-loaded with control HIV peptide. Data were analysed by the minimum χ^2^ method with 95% confidence intervals, and the CTL precursor frequency was calculated by Taswell's method (Taswell, 1981).

## RESULTS

### CTL-precursors in gastric cancer patients

PBMCs of cancer patients (*n*=80; 40 gastric, 15 colon, 11 lung, seven gynecological, three prostate cancers, and four melanomas) and healthy donors (*n*=31) were tested for their reactivity to a corresponding peptide after stimulation with each peptide. Results regarding HLA-A24^+^ (*n*=20) and -A2^+^ (*n*=20) gastric cancer patients are shown in Tables 1 and 2, respectively. PBMCs from 16 of 20 HLA-A24^+^ gastric cancer patients possessed CTL-precursors reactive to at least one of 13 peptides of vaccine candidates tested (Table 1). Among these 16 patients, five, one, three, six and one patients had CTL-precursors reactive to one, two, three, four and five peptides of vaccine candidates, respectively. The mean number of positive peptides was 2.8 per patient. Seven and six patients had CTL-precursors reactive to ART4_75_ and SART3_109_ peptides, respectively. Four patients had detectable levels of CTL-precursors to SART2_899_, SART3_315_, lck_208_ and lck_488_ peptides. CTL-precursors to the other seven peptides were also detectable in several patients. The profile of positive peptides entirely varied in 16 patients. In contrast, CTL-precursors reactive to these peptides were undetectable in PBMCs of the remaining four cancer patients. It is of note that CTL-precursors reactive to either Flu- or EBV-peptide, taken as positive control peptide, were also undetectable in PBMCs from those four cancer patients.

PBMCs from 11 of 20 HLA-A2^+^ gastric cancer patients possessed CTL-precursors reactive to at least one of 15 peptides of vaccine candidates tested (Table 2). Among them, five, one, three, one and one patients had CTL-precursors reactive to one, two, three, four and seven peptides, respectively. The mean number of positive peptides was 2.3 peptides per patient. Four patients had CTL-precursors reactive to HNRPL_140_ peptide, while three patients had those reactive to SART3_302_ and SART3_309_ peptides. CTL-precursors were also found to the others except one (ppMAPkkk_432_) peptide with lower frequencies. The profile of positive peptides was also entirely different among 11 patients. In contrast, CTL-precursors reactive to these peptides were undetectable in the remaining nine patients. Among them, seven patients had no detectable level of CTL-precursor reacting to either Flu- or EBV-peptide.

### Cytotoxicity of peptide-induced CTLs

**Figure 1:**
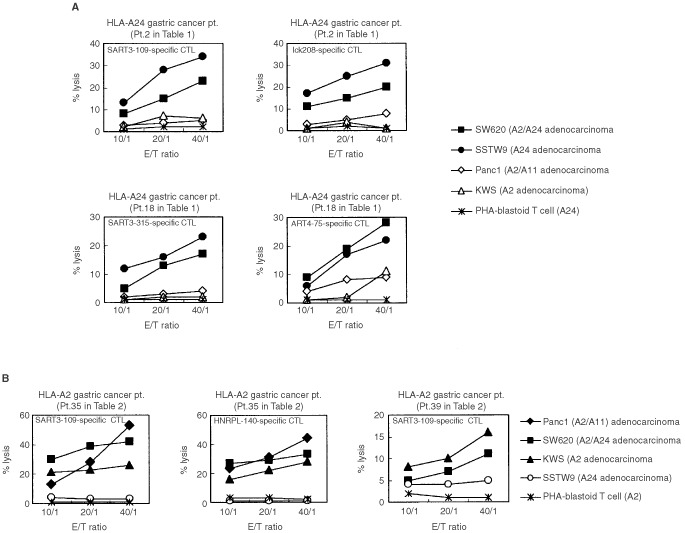
Peptide-induced CTL activity. PBMCs stimulated with peptides were expanded in the presence of feeder cells for 21–25 days, and were tested for their cytotoxicity against various target cells by a 6 h ^51^Cr-release assay. Representative results of HLA-A24^+^ and -A2^+^ patients are shown in (**A**) and (**B**) respectively.

A standard 6 h ^51^Cr-release assay was employed to confirm the anti-tumour response of the peptide-induced CTLs in four cancer patients. PBMCs of Pt 2 (HLA-A24^+^) showed significant levels of cytotoxicity against HLA-A24^+^ tumour cells (SW620 and SSTW9), but not against HLA-A24^−^ tumour cells (Panc1 and KWS) or normal PHA-blastoid T cells, when they were stimulated with each of the positive peptides derived from cancer-related antigens (SART3_109_, lck_208_, and lck_488_) (Figure 1A

and Table 3

**Table 3:**
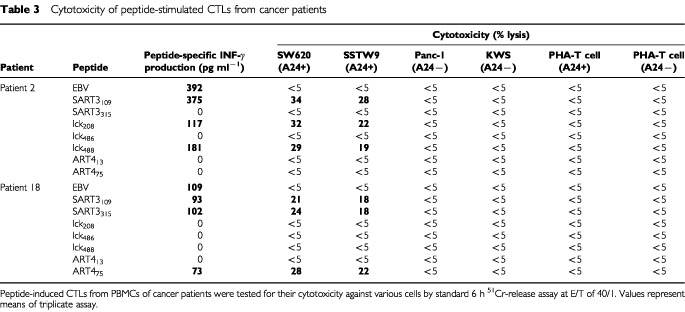
Cytotoxicity of peptide-stimulated CTLs from cancer patients

). In contrast, those stimulated with either EBV peptide or any of the negative peptides (SART3_315_, lck_486_, ART4_13_, and ART4_75_) were not cytotoxic to any of the target cells tested. Similar results were obtained in the PBMCs of Pt 18 who had CTL-precursors reactive to EBV, SART3_109_, SART3_315_, and ART4_75_ (Figure 1A and Table 3). PBMCs of Pt 35 (HLA-A2^+^) possessed CTL-precursors reacting to Flu, EBV, SART3_302_, HNRPL_140_ and the other five peptides (Table 2). Those PBMCs stimulated with SART3_302_ and HNRPL_140_ also showed HLA-A2-restricted and tumour-specific CTL activities (Figure 1B), whereas those stimulated with Flu, EBV, or any of the negative peptides had no CTL activity (data not shown). Similarly, PBMCs of Pt 39 had CTL-precursors reactive to Flu, EBV, and HNRPL_501_ peptides, and those stimulated with HNRPL_501_ peptide (Figure 1B), but not those with the other peptides (Flu, EBV, or negative peptides), showed HLA-A2-restricted and tumour-specific CTL activity. These CTL activities were inhibited by either anti-class I or -CD8 monoclonal antibody (mAb), but not by the other mAbs tested (data not shown).

### CTL precursor frequency analysis

**Figure 2:**
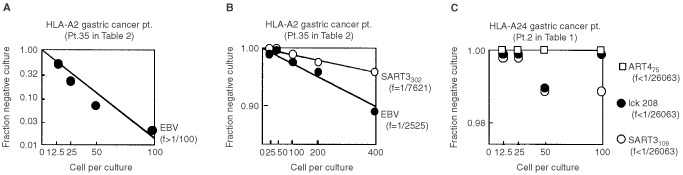
Precursor frequency analysis by limiting dilution method. PBMCs of a cancer patient (Pt 35) cultured with EBV peptide for 10 days by the new culture method (**A**), unstimulated PBMCs of the same patient (**B**) and unstimulated PBMCs of another cancer patient (Pt 2) (**C**) were served for limiting dilution culture as described in Materials and Methods. Cells from each well were tested at 9 to 15 days of culture for IFN-γ production in the presence of target cells. Wells were considered positive if they contained effector cells producing much higher level (>100 pg ml^−1^) and also statistically significant levels (*P*<0.05 by two-tailed Student-*t* test) of IFN-γ in response to CIR-A2402 or T2 cells pre-loaded with a corresponding peptide as compared with IFN-γ levels in response to CIR-A2402 or T2 cells pre-loaded with HIV peptide in duplicate assay. Data were analysed by the minimum χ^2^ method with 95% confidence intervals, and the CTL precursor frequency was calculated by Taswell's method.

A standard CTL precursor frequency analysis by limiting dilution method was performed in certain cases to compare the results obtained by the new culture method for CTL-induction. Representative results are shown in Figure 2

. PBMCs of Pt 35 stimulated with EBV peptide by the new method were provided as a positive control at 400, 200, 100, 50, 25 and 12.5 cells per well for CTL-precursor frequency analysis in duplicate assay. CTL-precursors were detectable in all 96 wells when cells were harvested from wells of 400 and 200 cells per well. CTL-precursors were detectable in 94, 88, 77 and 30 wells when cells were harvested from wells of 100, 50, 25 and 12.5 cells per well (Figure 2A). Subsequently the CTL-frequency was evaluated as at least >1 out of 100. Unstimulated PBMCs from the same patient were directly provided for CTL-precursor analysis in response to EBV and SART3_302_ peptide to which CTL-precursors were detectable by means of our culture method (Pt 35 in Table 2). Precursor frequencies of EBV and SART3_302_ peptide-specific CTL were found to be one out of 2525 and one out of 7621, respectively (Figure 2B). Unstimulated PBMCs of Pt 2 were also provided for CTL-precursor analysis in response to SART3_109_, lck_208_ and ART4_75_ peptides. CTL-precursors to any of these three peptides were under detectable levels (<1 out of 26 063) in this precursor frequency analysis. However, CTL-precursors reactive to the positive peptides (SART3_109_ and lck_208_) were found in one or two wells and no positive well was detected for the negative (ART4_75_) (Figure 2C).

### CTL-precursors in other cancer patients and healthy donors

The results of the induction assay regarding cancer patients other than those with gastric cancer are shown in Table 4

**Table 4:**
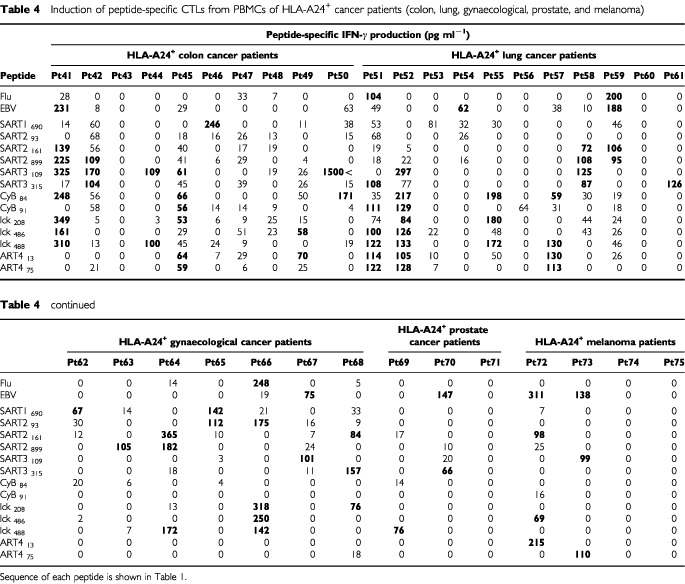
Induction of peptide-specific CTLs from PBMCs of HLA-A24^+^ cancer patients (colon, lung, gynaecological, prostate, and melanoma)

(HLA-A24^+^ 10 colon, 11 lung, seven gynecological, three prostate cancer, and four melanoma patients) and in Table 5

**Table 5:**
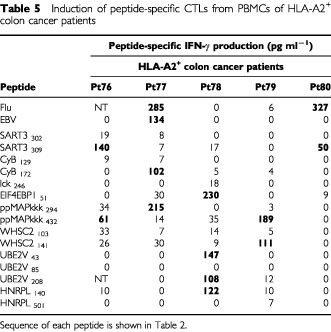
Induction of peptide-specific CTLs from PBMCs of HLA-A2^+^ colon cancer patients

(HLA-A2^+^ five colon cancer patients). The same CTL-induction assay was also performed for 31 healthy donors (20 HLA-A24^+^ donors and 11 HLA-A2^+^ donors). Overall reactivity to each peptide in all 80 cancer patients and in 31 healthy donors is shown in Table 6

**Table 6:**
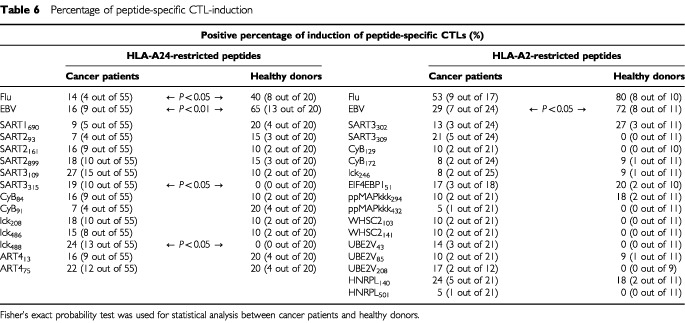
Percentage of peptide-specific CTL-induction

. CTL-precursors reactive to at least one of the vaccine-candidate-peptides were detectable in 57 out of 80 patients (71%), and the mean numbers of positive peptides were 2.0 peptides per positive case (ranging from 1–8 peptides). A profile of the positive peptides of vaccine candidates varied among these 57 patients, and seemed not to be influenced by the cancer origin. CTL-precursors reacting to cancer-related peptides were also detectable in 20 out of 31 healthy donors (64%), and the mean number of positive peptides in each donor was 1.4 (ranging from 1–4 peptides).

The percentages of detection of CTL-precursors reacting to foreign antigen peptide (Flu and EBV) in cancer patients were significantly lower than those in healthy donors in HLA-A24 cases (Table 6, left column). Those of EBV-specific CTL-precursors in cancer patients were also significantly lower than those in healthy donors in HLA-A2 cases (Table 6, right column). In contrast, the percentages of cases having CTL-precursors reactive to SART3_315_ and lck_488_ peptides in HLA-A24^+^ patients were significantly higher than those in HLA-A24^+^ healthy donors (Table 6, left column). A similar trend was also observed in certain WHSC2- and UBE2V-derived peptides in HLA-A2^+^ patients (Table 6, right column).

## DISCUSSION

We have demonstrated in the present study that peptide-specific CTL-precursors are detectable in the majority of cancer patients, and that PBMCs incubated with the positive peptides, not with the negative peptides, show significant levels of HLA-class-I-restricted cytotoxicity to cancer cells. These results suggest that vaccination of the positive peptides to cancer patients induces more potent anti-tumour response than that of the negative peptides.

Twenty-eight peptides (13 for HLA-A24^+^ and 15 for HLA-A2^+^ cancer patients) were used in this study. There are, however, many other peptides that are vaccine candidates having the ability to induce HLA-class-I-restricted CTLs reactive to cancer cells. Therefore, increased numbers of peptides for the assay should lead to an increase in the number of patients having CTL-precursors reactive to peptides and in the number of positive peptides per patient. These two HLA-class I alleles (HLA-A24 and -A2) are observed in >70% of Caucasians, >80% of Asians, and >40% of Blacks (Imanishi *et al*, 1992; Tokugawa *et al*, 1997). The present study also showed that the profile of positive peptides varies among patients, and that there is no obvious correlation regarding the types of cancers tested (gastric, colon, lung, gynaecological, and prostate cancers, and melanomas) or clinical stages (presence or absence of distant metastasis). Therefore, CTL-precursors reactive to peptides of vaccine candidates could be detectable in PBMCs of a large number of cancer patients throughout the world, regardless of cancer origin or clinical stage.

It is important to determine whether or not detection of peptide-specific CTL-precursors was associated with the expression of corresponding antigen in individual cancer patients. However, most epithelial cancer patients enrolled in this study were inoperable and availability of tumour samples was very limited in contrast to melanoma patients. In regard with SART-1, -2, -3, and ART4 antigens, we previously reported their protein expression in many samples of the majority of epithelial cancer cells and tissues by Western blot and Northern analyses (Shichijo *et al*, 1998; Yang *et al*, 1999; Nakao *et al*, 2000). We also reported that lck antigen was expressed in the majority of metastatic cancer cells and tissues (Harashima *et al*, 2001).

Difference in affinities of the peptides to the HLA-class I molecules might affect the efficacy of *in vitro* sensitization of PBMCs from cancer patients. In regard to peptides derived from either SART2 or SART3, their scores, an estimated half-time dissociation determined by computer program (Parker *et al*, 1994), were compared and it was found that peptides with higher scores seemed to induce peptide-specific CTLs more efficiently than those with lower scores (unpublished observation).

PBMCs from healthy donors also had CTL-precursors to peptides of vaccine candidates with relatively lower frequency, which was expected from our previous results showing that all the tumour-rejection antigens, from which the peptides originated, used in this study are non-mutated self-antigens preferentially expressed in proliferating cells, including malignant and normal cells (Yang *et al*, 1999). However, CTLs induced by these peptides showed cytotoxicity against cancer cells, but not against normal proliferating cells, as also demonstrated previously (Yang *et al*, 1999). Therefore, vaccination of these peptides may not be associated with adverse events in normal cells and normal tissues. Indeed, no severe adverse events have been observed in phase I clinical studies at the Kurume University Hospital, where 13 different peptides, also used *in vitro* in this study, have been used for HLA-A24^+^ cancer patients as peptides vaccines *in vivo* (Miyagi *et al*, 2001, and the other unpublished data).

Peptide-specific CTL-precursors were detected in healthy donors. One might wonder whether CTL-precursors of cancer patients are distinguishable from those of healthy donors or not. We observed that CyB peptide-stimulated CTLs from cancer patients had a tendency to show both peptide-specific and tumour-reactive responses, but those from healthy donors seemed to exhibit peptide-specific responses but no tumour-reactive response (Gomi *et al*, 1999). We suppose that, in healthy donors, CTL-precursors reacting to CyB peptides were primed as a result of cross-reactivity of CyB to bacteria-derived exogenous antigens (Ohkouchi *et al*, 2002). We also repeatedly reported that peptide-stimulated PBMCs from certain healthy donors produced significant levels of IFN-γ in response to peptides, but rarely showed cytotoxicity against tumour cells in an HLA-A24 or -A2 restricted manner by means of a ^51^Cr-release assay (Harashima *et al*, 2001; Ito *et al*, 2000, 2001; Kawano *et al*, 2000; Nishizaka *et al*, 2000). In addition, peptide-specific CTLs from cancer patients proliferated well for a long time in culture with IL-2 alone and thus these expanded cells became available in use for a ^51^Cr-release assay, whereas those from healthy donors did not (unpublished data). Therefore, measurement of CTL activity with a standard ^51^Cr-release assay could be a good tool to distinguish CTL-precursors of cancer patients from those of healthy donors.

There is a possibility that vaccination with a given peptide could result in *in vivo* immunoselection of host tumour *in situ,* and that further boosting against such epitopes might be unsuccessful. Jager *et al* (1996) reported an inverse correlation of antigen expression and CTL response in patients with metastatic melanoma (Jager *et al*, 1996). However, as described in the Introduction, we undertook this study to determine whether CTL precursors were detectable at the prevaccination state, based on the idea that boosting of circulating CTL precursors could be more reasonable than newly priming of CTLs. We suppose that an appropriate clinical trial is needed to answer to this key question. A phase I study of CTL-precursor-oriented vaccine, in which prevaccination PBMCs were screened *in vitro* for their reactivity to each of the peptides followed by vaccination of only the positive peptides, has been in progress since November 2000 at Kurume University. The initial study of immune responses to both peptides and tumour cells in post-vaccination PBMCs of colorectal cancer patients suggested that the present regime could be superior to the conventional regime to elicit prompt and strong immune responses.

CTL-precursors detected by this new culture method were also detectable by the standard CTL-precursor analysis in the Pt 35 case, but not in the Pt 2 case. Therefore, the limit of sensitivity of the new method may be higher than that of the conventional CTL-precursor frequency analysis. PBMCs were stimulated five or 0 times before the assay by the new method or by CTL-precursor frequency analysis, respectively, which might have influenced the limit of sensitivity. The limit of sensitivity of the standard CTL-precursor analysis is one out of 26 063 (Taswell, 1981), whereas the limit of sensitivity of the new method used in this study is one out of 100 000 (Hida *et al*, 2002). The specificity and positive predictive value of the CTL induction system used in this study were found to be high enough for clinical use (90 and 100%, respectively) (data not shown). The CTL induction method used in this study also has some other advantages compared to the standard CTL-precursor analysis or the tetramer method. The cost of the standard CTL-precursor analysis is more than 10 times that of the method used in this study (approximately $500 *vs* $30, per peptide, respectively), and the standard CTL-precursor analysis requires large number of feeder cells so that the influence of allogeneic feeder cells may not be entirely excluded. Although a tetramer assay is an alternative method, it does not directly reflect the functional activity of CTLs such as that reflected by cytotoxicity or cytokine production.

In conclusion, this study has showed that peptide-specific CTL-precursors are detectable in PBMCs of cancer patients prior to vaccination, thus providing a scientific basis for the development of a CTL-precursor-oriented peptide vaccine as an order-made cancer immunotherapy for the majority of cancer patients throughout the world. The same vaccine strategy may be applicable to patients infected with HIV or other pathogenic viruses, for which no effective vaccine is available.
